# Drive: Theory and Construct Validation

**DOI:** 10.1371/journal.pone.0157295

**Published:** 2016-07-13

**Authors:** Alex B. Siegling, K. V. Petrides

**Affiliations:** London Psychometric Laboratory, University College London, London, United Kingdom; IRCCS Istituto Auxologico Italiano, ITALY

## Abstract

This article explicates the theory of drive and describes the development and validation of two measures. A representative set of drive facets was derived from an extensive corpus of human attributes (Study 1). Operationalised using an International Personality Item Pool version (the Drive:IPIP), a three-factor model was extracted from the facets in two samples and confirmed on a third sample (Study 2). The multi-item IPIP measure showed congruence with a short form, based on single-item ratings of the facets, and both demonstrated cross-informant reliability. Evidence also supported the measures’ convergent, discriminant, concurrent, and incremental validity (Study 3). Based on very promising findings, the authors hope to initiate a stream of research in what is argued to be a rather neglected niche of individual differences and non-cognitive assessment.

## Introduction

In contrast to intelligence and personality (the other main pillars of individual differences), the substantive literature on motivation can be described as ‘fuzzy’ at the conceptual and taxonomic levels, lacking organisational frameworks to accommodate the multitude of constructs involved [1]. Motivation in the context of individual differences and psychological assessment is concerned with two fundamental questions about human behaviour: (a) *why*? (specific personal reasons for one’s behaviour) and (b) *how much*, *or to what extent*? (the propensity to pursue personal goals, motives, needs, etc.) [2,3].

The present article draws an explicit conceptual distinction between motivational level (referred to as *drive*) and motivational reasons (referred to as *motivators*), which is argued to be a systematic omission in the individual differences and assessment literature of motivation. Motivators reflect what a person wants to attain, or the ulterior reasons for one’s behaviour (interests, goals, preferences, needs, attitudes, desires, etc.), whereas drive concerns the extent to which a person acts on personal motivators, whatever these may be. The article then proposes a theory of drive and proceeds to a basic construct validation programme. On the basis of two measures, questions of substantive and structural validity were examined systematically, whereas aspects of external validity (convergent, discriminant, and criterion) were examined on a preliminary basis. Prior to this, the literature concerning individual differences in, and the assessment of, motivation is briefly reviewed in order to anchor the present work.

### Motivation: Individual Differences and Measurement

The vast majority of measures claimed to assess motivation, or some aspect of it, focus on motivators, such as motives (e.g., success, power, and affiliation) and cognate families of constructs, such as interests, goals, and attitudes [1,2]. Examples of measures assessing sets of multiple, universally relevant motivators, often containing the “big three” (affiliation, power, and achievement), are the Personality Research Form [4], the Reiss Profile [5,6], the projective Thematic Apperception Test [7], the Motives, Values, and Preferences Inventory [8], and the Unified Motives Scales [9]. Specific area measures assess multiple motivators relevant to a particular context, including work [10], academia [11], or athletics [12]. Narrower area measures focus either on a very specific motivational area [13] or on a single motivator [14–16].

[8] emphasised that the families of concepts incorporated here under the umbrella term “motivators” (motives, values, preferences, interests, etc.) are closely related and often used interchangeably; they differ mainly in their breadth and level of abstraction. Recent empirical support for the integration of these cognate families of concepts comes from the integration of available measures into the Unified Motives Scales [9].

Another range of measures focuses on the mechanisms underlying individual differences in motivation and on what [1] have termed “motivational dynamics” (how motives are integrated within one’s mental life). Examples include the distinctions of intrinsic motivation (from within) versus extrinsic motivation (via external incentives) [17], mastery orientation (desire to acquire new knowledge or skills) versus performance orientation (desire to demonstrate ability and impress) [18], as well as approach motivation (engagement rewarded by positive outcomes) versus avoidance motivation (disengagement rewarded by positive outcomes) [19]. These measures focus on the types of reasons for, and nature of, people’s behaviour, rather than particular motivators or drive specifically. Still, some of them are intertwined with particular motivational areas [17].

Relatively little attention has been paid to what the lay person would also associate with motivation: the *how much* and *to what extent* a person does what he or she wants to do (i.e., drive). Ceommon dictionary definitions, for instance, concern the propensity to be motivated as much as they bear on ulterior reasons for behaviour [20,21]. Consequently, measures of motivational level (drive) that are not contaminated by motivators of some form are hard to find. For example, the Motivation and Engagement Scale [22], although conceptually reflective of drive, integrates motivator-like concepts, such as failure avoidance, mastery orientation, and uncertainty control, along with a few facets representative of drive (e.g., persistence, task management, and planning). An exception is the Motivation and Energy Inventory [23], which focuses on current motivational level, or states (operationalised as mental energy, physical energy, and social motivation) rather than any form of motivator.

Other measures seem to tap into the construct (drive) partially. Yet, they would be concealed as measures of other constructs, possibly because their deviation from motivators does not fit into the mainstream literature, or perhaps to emphasise the novelty of the measure. Candidates include grit, amotivation, or goal pursuit. For example, Duckworth’s Grit Scale consists of the subscales passion and perseverance [24,25], two “non-motivators” that are highly reflective of drive. However, grit has not been linked to the substantive motivation area (e.g., the term *motivation* that overarches drive and motivators does not appear in the development article of this scale).

### Drive: Theory and Nomological Network

There are good reasons to draw an explicit distinction between drive and motivators. As discussed, motivators are the personal reasons for what a person wants or does, whereas drive describes the extent of the person’s investment and is more similar to energy. Imagine two people who, due to similar upbringings and social backgrounds, have the same life goals (motivators) but who differ in their drive to realise them. Having a certain motivator does not necessarily entail the drive to act on it, or else we would have many more aspiring millionaires and gold medallists. Furthermore, when we talk about our level of motivation for various tasks (i.e., drive), we do not imply that the ulterior motivator for our behaviour is low or high. This conceptual difference has been implicitly recognised in the analogous distinction between goal content, which fits with motivators, and goal pursuit, which is more akin to drive [26].

Another reason for an explicit distinction concerns the diversity and multidimensional structure of various types of motivators. Factor analytic research shows that multiple distinct dimensions underlie individual differences in these interrelated families of constructs [4–6,27,28]. This finding is in line with theory, for there is no reason why having particular motivators (e.g., recognition, power, or hedonistic motives) should imply a greater likelihood of having any other motivators (e.g., aesthetic or scientific motives). Accordingly, the plural form (e.g., motives, interests, goals, desires) is typically used in the label of relevant scales and in the wider literature to refer to these families of attributes. In contrast, there is no reason for drive to be multidimensional, or to consist of orthogonal and even modestly related constructs. Evidence from similar constructs, such as “grit” (passion and perseverance for long-term goals), amotivation (lacking motivation to engage and perceived competence, and failing to see value in an activity), and goal pursuit, provides empirical support for an individual superordinate construct. This is not to say, of course, that one cannot be more motivated to engage in one type of task (e.g., schoolwork) over another (sport). Here, it is simply argued that drive and motivators should be measured independently because they are psychologically distinct, not unrelated.

Drive can be conceptualised as permeating personality-factor space [29], concerning typical patterns in affect, behaviour, and cognition. In that respect, drive has cross-situational as well as temporal stability, although inevitably linked to motivational states (i.e., variations in motivational level across time and situations). Personality concerns *how* a person is like and behaves, whereas drive describes *how much*, or to *what extent*, he/she does something in terms of commitment, sacrifice, effort, etc. As such, drive bears particular resemblance to those personality dimensions that involve considerable effort and self-control, such as Conscientiousness and Neuroticism (Emotional Stability) [30]. Indeed, associations of similar constructs (e.g., grit) with the Big Five would suggest associations with, primarily, Conscientiousness and, secondarily and negatively, Neuroticism [25]. Still, another part of the construct is theorised to be specific, or unrelated to the established personality dimensions. Drive facets should, therefore, occupy a distinct dimension in personality-factor space, even if cross-loadings are to be expected.

The role of motivation in personality, particularly in the context of the Big Five personality traits, was previously examined and discussed by [31]. Taking a multidimensional stance, they defined Openness (to experience), for example, as individual differences in the activation of the reward system during active cognitive processing, and Agreeableness as differences in the motivation to cooperate during resource conflicts. If motivational components implicit in broad personality dimensions intercorrelate, a general mechanism may well run across these dimensions and contribute to a multidimensional range of behaviours, although its activation may vary between individuals across domains [32]. Consistent with a unidimensional view is the conceptualisation of motivation as a process of seeking (tendency to approach intellectually challenging situations) and conquering (tendency to master those challenging situations) in the context of intellect, intrinsic motivation, and curiosity [33]. These highly interrelated factors (*r* = .86) both correlated with multiple personality dimensions, foremost Openness/Intellect (*r* = .72, .71) and Conscientiousness (*r* = .20, .47).

Given its theoretical basis, drive can be expected to offer explanatory gains in numerous contexts (e.g., education, work, and leisure) and for several important life outcomes (e.g., achievement, success, and societal contribution). It would then qualify as having utility in various assessment applications, especially in occupational and educational settings. Drive would even seem to be implicated in maladaptive outcomes such as depression (at the low extreme), which often involves a severe lack of drive (e.g., getting out of bed), and mania (at the high extreme), characterised by excessive engagement [34]. While a person’s motivational level in any given situation (state motivation) is a theoretically more salient predictor of behaviour in that situation, drive shapes and predicts future motivational states, and thereby behaviour, across situations; it is a best estimate of motivational states in any given future situation.

This article describes the construct validation of drive, following [35]’s three-stage framework [36,37]. To this end, two measures, based on a systematically derived representation of the construct, were developed and validated in a series of three studies. In Study 1, a comprehensive corpus of human attributes [38] was examined by independent raters for theoretically relevant facets, which were subsequently evaluated by independent judges and reviewers. In Study 2, the facets were examined for their reliability and internal structure, using an extensive item measure of the facet scales. Moreover, cross-informant reliability and congruence with a short form, based on single-item ratings of the facets, were assessed. The external validity of the measures (i.e., convergent, discriminant, and criterion validities) was examined in Study 3.

## Study 1: Substantive Validity

Comprised of facets found in several well-established measures, the International Personality Item Pool (IPIP) [38] served as a corpus for identifying a comprehensive and initially overinclusive set of facets. Specific facet-level traits assessed with major personality inventories, and hence the IPIP, fit with a cross-situational and temporally stable conceptualisation of drive, representing typical behaviour, thoughts, and feelings; they are not exclusively representative of personality. Consider the facet of diligence included under the Conscientiousness domain in the HEXACO model of personality. Besides being conscientious, the diligent individual needs the drive to actually approach tasks in a diligent manner. Facet-level traits comprise variance unaccounted for by higher-order personality dimensions [39]. Some of that variance is theorised to be part of drive.

The IPIP provides an exhaustive set of “non-ability” attributes (245 at the time of this study), a subset of which is presumably representative of the drive dimension. It comprises the facets of several established personality inventories and, therefore, extends beyond the facets used to represent and operationalise the Big Five personality traits.

### Method

Two raters undertook an initial screening of the IPIP facets. The derived overinclusive set of facets was then scrutinised by a group of judges with psychology backgrounds and finally reviewed by several subject-matter experts.

#### Raters and judges

The two initial raters of the IPIP facets (one male, one female) were a researcher involved in the investigation and a trained research assistant. The researcher was completing his PhD in psychology and had specific knowledge in the subject area. The research assistant, a Masters-level manager of a university library with experience in staff management and a psychology background, was well briefed on the subject area.

A group of judges were five PhD students and one post-doc in Psychology (three female, three male) from diverse cultural backgrounds (Turkish/British, two British, Canadian/Lebanese, Italian, and Canadian). Three of them had a research and practitioner focus, while the other three were purely focused on research at the time. Subject-matter experts were five academics in the field. Background information were not collected from this group.

#### Procedure

Determining the indicators of higher-order constructs necessitates a clear set of inclusion criteria [40]. Based on the theory of drive, three criteria were explicated: (a) indicators should represent the construct (motivational level, or drive) and not be a motivator (e.g., interests, motives, goals, needs); (b) indicators should be facets situated at the same level of abstraction (the narrowest trait level) and not higher-order constructs, antecedents of the construct, outcomes, or very specific expressions typically captured by individual items; and (c) facets should be context-independent. Criterion B and largely also Criterion C were addressed by using the IPIP as the source of facets, since it consists of general facet-level traits. Criterion A was discussed in detail with the raters and judges.

Once it was clear that both raters had understood the meaning of drive and its distinction from motivators, they independently selected relevant facets from the IPIP list. They were asked to include facets about which they were not entirely sure, given subsequent refinement procedures. Raters were allowed to look up the meaning of any adjectives in a general language dictionary; specific definitions were not provided. The judges were asked to evaluate the selected facets with respect to the construct definition and explanation. Since the judges were dispersed geographically, they completed the task online, with all instructions (including construct definition) presented in written form. They were asked to rate each of the selected attributes using the following options: “Yes–motivation”, “Maybe–not sure”, or “No–not motivation”. Instructions referred the judges to an electronic dictionary in case they needed to look up the meaning of any facets they were not sure about. Reviews by subject-matter experts were unstructured and qualitative. They could say as much or as little as they wanted about the facets.

### Results and Discussion

The inter-rater reliability among the initial raters was strong (Cohen’s κ = .88) and 24 facets were identified. Disagreement only occurred in five instances: prudence, temperance, self-efficacy, self-control, and self-confidence. To ascertain that the preliminary set of facets would be overinclusive, rather than underrepresentative [37,41], facets selected by only one of the two raters were retained at this stage, along with those on which both agreed.

There were no facets that none of the judges endorsed (by indicating “Yes”). “Yes” was the most frequent selection by the judges in almost all instances. The only exception was joyfulness, where four judges said “maybe–not sure” and two said “Yes”. The “No–not motivation” option was typically selected by one or two judges or none at all. In only one case (self-control), three judges selected this option (as many as those who said “Yes”). Given these results, none of the 24 attributes identified by either or both of the initial two judges were dropped at this stage. However, two of the five expert reviewers indicated that the facets competence and self-efficacy are distinct and should not be included in the measure, because both primarily concern the real and perceived abilities to do things, rather than motivation. It was agreed that these two facets stand out conceptually and, therefore, the decision was made to discard them at this stage. Additionally, the authors felt that the meaning of another facet, activity-level, was too ambiguous, especially compared to the other facets, which have more specific meaning. Thus, the 21 facets shown Table 1 were retained for Study 2.

**Table 1:** Internal Reliabilities of IPIP Facet Scales and Corrected Facet-Total Correlations in Study Samples.

Facet (no. of items)	Online sample (*N* = 362)		British sample (*N* = 241)		Eugene-Springfield community sample (*N* = 496)	
	Cronbach’s α	Corrected facet-total correlation	Cronbach’s α	Corrected facet-total correlation	Cronbach’s α	Corrected facet-total correlation
Self-confidence (5)	0.78	0.74	0.70	0.64	0.70	0.67
Temperance (9)	0.77	0.62	0.67 (0.78)	0.54	0.73	0.50
Zest/enthusiasm/vitality (9)	0.82	0.78	0.78	0.76	0.79	0.63
Valour/bravery/courage (10)	0.80	0.56	0.78	0.49	0.75	0.51
Liveliness (8)	0.85	0.72	0.83	0.66	0.82	0.57
Insight (7)	0.76	0.64	0.75	0.64	0.71	0.52
Initiative (5)	0.81	0.73	0.81	0.67	0.74	0.53
Diligence (5)	0.70	0.78	0.69 (0.80)	0.73	0.66 (0.78)	0.61
Deliberateness (6)	0.69 (0.79)	0.48	0.61 (0.74)	0.50	0.60 (0.75)	0.39
Competitive (5)	0.66 (0.79)	0.74	0.59 (0.76)	0.75	0.64 (0.78)	0.72
Experience-seeking (5)	0.69 (0.81)	0.48	0.65 (0.79)	0.37	0.61 (0.77)	0.36
Generates ideas (5)	0.85	0.44	0.81	0.52	0.78	0.52
Prudence (7)	0.75	0.53	0.71	0.52	0.69 (0.79)	0.52
Resourcefulness (6)	0.75	0.84	0.69 (0.81)	0.76	0.75	0.72
Self-control (9)	0.71	0.29	0.71	0.20	0.67 (0.78)	0.13
Ind./persev./persis. (7)	0.81	0.75	0.77	0.65	0.80	0.64
Adventurousness (7)	0.73	0.42	0.78	0.35	0.66 (0.77)	0.33
Self-discipline (5)	0.77	0.76	0.70	0.70	0.70	0.66
Achievement-striving (6)	0.71	0.72	0.75	0.69	0.64 (0.77)	0.62
Hope/optimism (8)	0.81	0.75	0.70	0.74	0.71	0.63
Joyfulness (7)	0.86	0.75	0.79	0.69	0.76	0.59

Where Cronbach’s alpha is low (< .70), McDonald’s omega is given in parentheses. IPIP = International Personality Item Pool; Ind./persev./persis. = Industriousness/perseverance/persistence.

## Study 2: Internal Validity

The focus of this study was [35]’s structural validity stage: the internal psychometric properties of the measure, viz. reliability and factor structure. Since no particular structural model could be determined on theoretical grounds, a hierarchical effect-indicator model was empirically derived from the facets, involving possible refinement of the construct representation. The factor structure was derived and tested using three samples, based on an IPIP measure of the facets. Further psychometric analyses (composite internal reliability, measurement congruence, and cross-informant reliability) involved a short form, an additional fourth sample, and informant-ratings for two of the samples.

### Method

The research reported in all five studies was approved by the UCL Research Ethics Committee (Project ID Number: CEHP/2014/525). Participants gave their informed consent by reading the document approved by the institutional research ethics board and indicating their agreement to these terms electronically.

#### Participants

All sample descriptions in this investigation (Studies 2 and 3) concern the valid cases only. “Bad cases” (e.g., partial responders, unrealistically fast responders of less than 2 average seconds per item, and those skipping numerous items) were removed from the datasets of three of four samples collected specifically for this investigation.

An Online sample was recruited using a mix of procedures. First, a recruitment notice was posted on recruitment platforms for academic research in psychology and on a general commercial recruitment platform for online academic research. Third, a recruitment notice was posted on Twitter by a prominent, non-academic authority and writer in the area of motivation. The total sample size was 362 participants (78.2% female), who had a mean age of 33.49 years (*SD* = 13.5, range = 15.9–73.1). Ethnic backgrounds of the participants were 76.1% Caucasian, 4.5% African, 3.6% Chinese, 3.9% South Asian (Bangladesh, India, and Pakistan), and 11.9% other or mixed. Educational qualifications obtained were distributed across GCSE/O or similar (5.8%), A Level or similar (19.9%), BA/BSc or similar (34.4%), MA/MSc or similar (19.6%), MBA (3.6%), PhD (2.5%), and other (14.1%). At the time of the study, 33.1% were enrolled in full-time and 8.8% in part-time education; 43.6% were working full-time and 18.0% part-time. As an incentive and token of appreciation, participants were entered into a price draw for one of several gift vouchers.

A British university sample (*N* = 241, 80.1% female) was recruited via an institutional subject pool. Most participants were full-time students (88.0%) and currently studying for a BA/BSc or similar degree (62.7%) or for an MA/MSc or similar degree (22.8%); other qualifications pursued included MBA, PhD, and other (each less than 3%). Relatively small proportions of the sample studied part-time and worked full-time or part-time (each less than 10%). The mean age of the sample was 22.4 years (*SD* = 6.7, range: 18.0 to 74.1 years) and 93% were below the age of 30. Participants were predominantly of Caucasian (49.8%) and Chinese (32.4%) ethnic backgrounds; the remainder came from South Asian (Pakistan, India, Sri Lanka; 3.3%), African (0.8%), and other or mixed backgrounds (13.7%). Most participants received course credit and all were entered into a price draw for gift vouchers.

It was also possible to use the data from the U.S.-based Eugene-Springfield community sample. The portion of the sample with complete data for the relevant IPIP scales comprised 496 adults (41.9% male) with a mean age of 50.7 years (*SD* = 11.8, range: 20 to 83 years). Virtually all of the participants were Caucasian (98.8%). The remaining were either Asian American, Hispanic, or other. The most prevalent educational levels were “some college” (28.5%), “post-college education” (25.4%), “college graduate” (20.5%), “high school graduate” (9.3%), and “some post-college education” (9.1%); others were “vocational/technical schooling” (5.7%) and “not graduated from high school” (1.4%). Participants were working full-time (44.6%) or part-time (14.9%), retired (19.6%), homemaker (9.0%), unemployed (3.1%), or did not specify their current role (8.1%).

A Norwegian sample (*N* = 142, 61.3% female) was recruited via Qualtrics Sample Finder. The mean age of this sample was 47.6 years (*SD* = 7.7) and participants reported an average of 33.8 years (*SD* = 9.5) of spoken English. The vast majority of the participants (96.5%) were of Caucasian descent. Highest educational qualifications were distributed across High school or similar (24.6%), BA/BSc or similar (22.5%), A Level, IB, or similar (19.7%), MA/MSc or similar (14.1%), MBA (9.9%), PhD (4.9%), and other (4.2%). Few participants (9.9%) were enrolled in part- or full-time education; approximately half the sample (47.9%) were full-time workers and another 21.1% worked on a part-time basis. This sample was financially compensated for their participation.

Subgroups of the Online (*n* = 46, 16 male) and British (*n* = 101, 28 male) samples nominated a person they thought would know them well enough. Informants were friends, family members, or romantic partners, who had known them for an average of 13.6 years (*SD* = 11.3) and 11.5 years (*SD* = 8.8), respectively.

#### Measures and procedure

An added advantage of using the IPIP as a gateway to operationalising drive is that it provides a uniform and validated set of items for every facet. Since modified versions of the respective IPIP scales were used to measure the 22 facets, this version will be referred to as Drive:IPIP. The Drive:IPIP scales and their respective items are in the public domain, available at ipip.ori.org. All rights and ownership reside with those who have developed the items and generously made them available for research. The measure is intended for research purposes in the general adult population.

A total of 33 items were removed from the facet scales, because several of the IPIP items appear in multiple facets. To maintain a conservative number of items per facet [42], 11 items included in two or more of the selected IPIP scales were retained for facets otherwise measured on the basis of only four items or less (without allowing any item in multiple facets). In total, 148 items were used to measure the 22 facets, ranging between 5 and 10 items per scale. This set of items can be found in S1 Appendix, organised by facets. The standard IPIP response scale, ranging from 1 (*very inaccurate*) to 5 (*very accurate*), was used.

The Drive:S uses direct estimates for each of the 8facets (i.e., single-item rating scales), with facet labels functioning as items. Single-item scales are an efficient and valid way to measure specific attributes [43]. Research comparing multi- and single-item scales has not revealed empirically observable differences in construct validity and methods variance [44]. Single-item ratings are particularly robust where positively worded Likert items are used [45] and the underlying construct is homogenous [46], which is the case here. The Online and British samples completed the items on a visual analogue scale, using an electronic slider (range: 0% to 100%). They were asked to rate themselves in comparison to other people of similar age. The Norwegian sample completed the items on a 7-point Likert scale, ranging from 1 (*very little*) to 7 (*very much*). The scale format was varied to examine any measurement effects linked to a particular scale format. The measure including instructions can be found in S2 Appendix.

The data from the Online, British, and Norwegian samples were collected via an electronic survey. After providing demographic information, these samples first completed the Drive:IPIP items followed by the Drive:S. Administered electronically, the order of items in these samples was randomised across participants. Missing responses were highlighted to these two samples, in case participants had skipped any items inadvertently.

#### Statistical analyses

Facet internal reliabilities, facet intercorrelations, and facet-total correlations were first examined in the three samples used to derive the factor structure. The purpose of facet-total correlations was to identify any obviously unrelated or “tangential” facets (*r* < .30) [47,48]. Next, facets were submitted to a Principal Component Analysis in the Online and British samples (two samples were used for exploratory purposes to minimise the risk of sample-specific effects impinging on the results). Preceding Exploratory Factor Analysis by an Principal Component Analysis is common practise [36,49] and had a twofold purpose: (a) to verify that the derived facets are homogenous and share a common dimension (i.e., the target construct) that accounts for a decent portion of their variance, and (b) to further assess the relevance of individual facets (and minimise the possibility of maintaining an overinclusive representation, since rather cautious selection procedures were employed in Studies 1 and 2).

Exploratory Factor Analysis, using principal axis factoring with oblique rotation (Promax method, delta = 4), was used to examine the facets for shared first-order factors. The Online and British samples were used for this purpose, given their relative diversity. The larger and presumably more homogenous Eugene-Springfield community sample was used to test the derived structure. In the first instance, model testing was performed via Confirmatory Factor Analysis with maximum-likelihood estimation. Since consensus on the use of fit indices including their cut-offs is lacking [50–54], it was decided to adopt a fluid approach focused on level of, rather than absolute, model fit. Commonly reported indices were used for this purpose: Comparative Fit Index (CFI), Root Mean Square Error of Approximation (RMSEA), and Standardised Root Mean Square Residual (SRMR). Yet, it is important to note that even clean structural models derived via Exploratory Factor Analysis often fail to achieve satisfactory fit in Confirmatory Factor Analysis for a majority of measures [55]. Thus, target rotation was reserved as an optional route to establishing structural reliability in the Eugene-Springfield community sample.

The last step was to examine internal reliabilities and bivariate correlations between the Drive:IPIP and Drive:S global composites, also involving cross-informant coefficients in the respective samples. Facet scores were used to compute internal reliabilities, since arbitrary variations in the number of items might lead to inaccurate coefficients.

### Results and Discussion

#### Internal reliability, correlations, and facet-total correlations

Internal reliabilities and corrected facet-total correlations for all three samples can be found in Table 1. Cronbach’s alphas were generally acceptable for each facet in at least one of the three samples. Where alphas were low (< .70), McDonald’s omega was computed [56] as an additional estimate of internal reliability (shown in parentheses in Table 1). Omega tends to be more accurate than alpha, which is a lower bound to internal reliability [56,57]. An inspection of intercorrelations among the facets showed that none of them were high enough (≥ .90) to consider merging any facets. Corrected facet-total correlations suggested that all except one of the facets are linked to the same dimension, the theoretical construct of drive; self-control showed consistently low correlations (< .30), with particularly low correlations (≤ .20) in two of the three samples. Consequently, this facet was excluded from further analyses.

#### Principal Component Analysis

Results for both samples are shown in Table 2. Although all facets loaded at least to some degree on the first component, four facets (prudence, deliberateness, experience-seeking, and adventurousness) had stronger loadings on the second ensuing component than on the first component in both samples, indicating that they share most of their variance with a dimension distinct from drive. Given the overinclusive selection procedures used in Study 1 and the multidimensional background of the derived indicators, it was decided to proceed without these four facets in further analyses.

**Table 2:** Principal Component Loadings of IPIP Facet Scales in the Online and British Samples.

Facet	Online sample (*N* = 362)				British sample (*N* = 241)			
	1	2	3	4	1	2	3	4
Self-confidence	0.78				0.70		0.30	
Temperance	0.62	-0.46	0.34		0.55	-0.50	0.46	
Zest/enthusiasm/vitality	0.82				0.81			
Valour/bravery/courage	0.63				0.55	0.35		0.31
Liveliness	0.77			-0.34	0.72			-0.43
Insight	0.68	0.49			0.69	0.48		
Initiative	0.77		-0.31		0.73	-0.36		
Diligence	0.82		-0.35		0.77	-0.41	-0.35	
Deliberateness	0.49	-0.65		0.42	0.50	-0.55	0.39	0.38
Competitive	0.79		-0.33		0.80		-0.31	
Experience-seeking	0.54	0.61			0.43	0.71		
Generates ideas	0.50	0.50		0.32	0.59	0.43		0.39
Prudence	0.54	-0.65		0.35	0.53	-0.63	0.33	
Resourcefulness	0.87				0.79			
Ind./persev./persis.	0.78	-0.42			0.69	-0.47		
Adventurousness	0.46	0.59		0.38	0.40	0.68		
Self-discipline	0.80	-0.33			0.75	-0.42		
Achievement-striving	0.77		-0.44		0.74		-0.45	
Hope/optimism	0.79		0.37		0.78			
Joyfulness	0.79		0.44		0.74		0.37	-0.31
**% of variance**	50.65	17.78	6.92	5.52	45.33	17.38	7.45	5.91

Factor loadings of < .30 are omitted from the table. IPIP = International Personality Item Pool; Ind./persev./persis. = Industriousness/perseverance/persistence.

The variance explained by the first component was near 50 percent in both samples. The fact that this component did not explain most of the facet variance is not unexpected, since the facets originate from multiple, largely distinct personality dimensions. In spite of incorporating variance otherwise distributed across different personality dimensions, a single dimension theorised to represent drive was clearly apparent in both samples. Overall, these results yield good preliminary support for the proposed construct, with all of the facets loading onto the same component.

#### Exploratory Factor Analysis

Analyses were conducted on the 16 remaining facets. In the Online sample, Kaiser’s criterion (of Eigenvalues greater than 1) and an unambiguous scree plot both indicated three factors. At this point, all three factors were identified by at least five loadings, but one facet (resourcefulness) cross-loaded on all three factors, while two other facets (temperance and competitive) loaded on two factors each. All cross-loadings were within .20 of the primary loading and therefore deemed critical. Dropping items with cross-loadings (within .20 specifically) is not uncommonly used as a rule-of-thumb in Exploratory Factor Analysis [48]. Undifferentiated cross-loadings indicate that a facet may be relatively broad in scope and possibly redundant with other facets. They also complicate model fitting in Confirmatory Factor Analysis. Upon discarding the three facets, no additional cross-loadings emerged. Consequently, a three-factor solution was accepted for the Online sample, the results of which are shown in Table 3. The three factors explained most of the facet variance and intercorrelated moderately.

**Table 3:** Pattern Matrix for Promax Three-Factor Solution Extracted from IPIP Facet Scales and Factor Correlation Matrix in the Online Sample.

Facet	Factor loading		
	1	2	3
Diligence	0.93		
Initiative	0.92		
Self-discipline	0.91		
Ind./persev./persis.	0.89		
Achievement-striving	0.76		
Joyfulness		1.12	
Hope/optimism		0.84	
Liveliness		0.82	
Zest/enthusiasm/vitality		0.73	
Self-confidence		0.61	
Insight			0.95
Generates ideas			0.86
Valour/bravery/courage			0.46
**Eigenvalue**	7.29	1.56	0.93
**% of variance**	56.08	11.98	7.12
**Factor**	**Factor correlations**		
1	—		
2	0.63	—	
3	0.47	0.60	—

*N* = 362. Factor loadings of < .30 are omitted from the table. IPIP = International Personality Item Pool; Ind./persev./persis. = Industriousness/perseverance/persistence.

In the British sample, analyses conducted on the 16 facets extracted three factors. The same three facets (resourcefulness, competitive, and temperance) that emerged as problematic in the Online sample again showed critical cross-loadings. As shown in Table 4, their removal resulted in a clean three-factor solution, identical to that extracted in the Online sample. The factors explained a similar amount of variance in the facet scores as they did in the Online sample and, again, showed intercorrelations of moderate strength.

**Table 4:** Pattern Matrix for Promax Three-Factor Solution Extracted from IPIP Facet Scales and Factor Correlation Matrix in the British Sample.

Facet	Factor loading		
	1	2	3
Diligence	0.95		
Ind./persev./persis.	0.91		
Self-discipline	0.89		
Initiative	0.85		
Achievement-striving	0.83		
Joyfulness		1.02	
Liveliness		0.93	
Hope/optimism		0.74	
Zest/enthusiasm/vitality		0.72	
Self-confidence		0.68	
Generates ideas			0.91
Insight			0.81
Valour/bravery/courage			0.78
**Eigenvalue**	6.84	2.12	1.16
**% of variance**	52.64	16.28	8.91
**Factor**	**Factor correlations**		
1	—		
2	0.51	—	
3	0.36	0.54	—

*N* = 241. Factor loadings of < .30 are omitted from the table. IPIP = International Personality Item Pool; Ind./persev./persis. = Industriousness/perseverance/persistence.

Aside from supporting a three-factor solution, the data of both samples showed the facets of resourcefulness, competitive, and temperance to be problematic due to undifferentiated cross-loadings. These particular facets also appear to be less conceptually reflective of drive than the 13 other facets. Therefore, the decision was made to exclude these facets from further analysis.

#### Model testing

**Fig 1:**
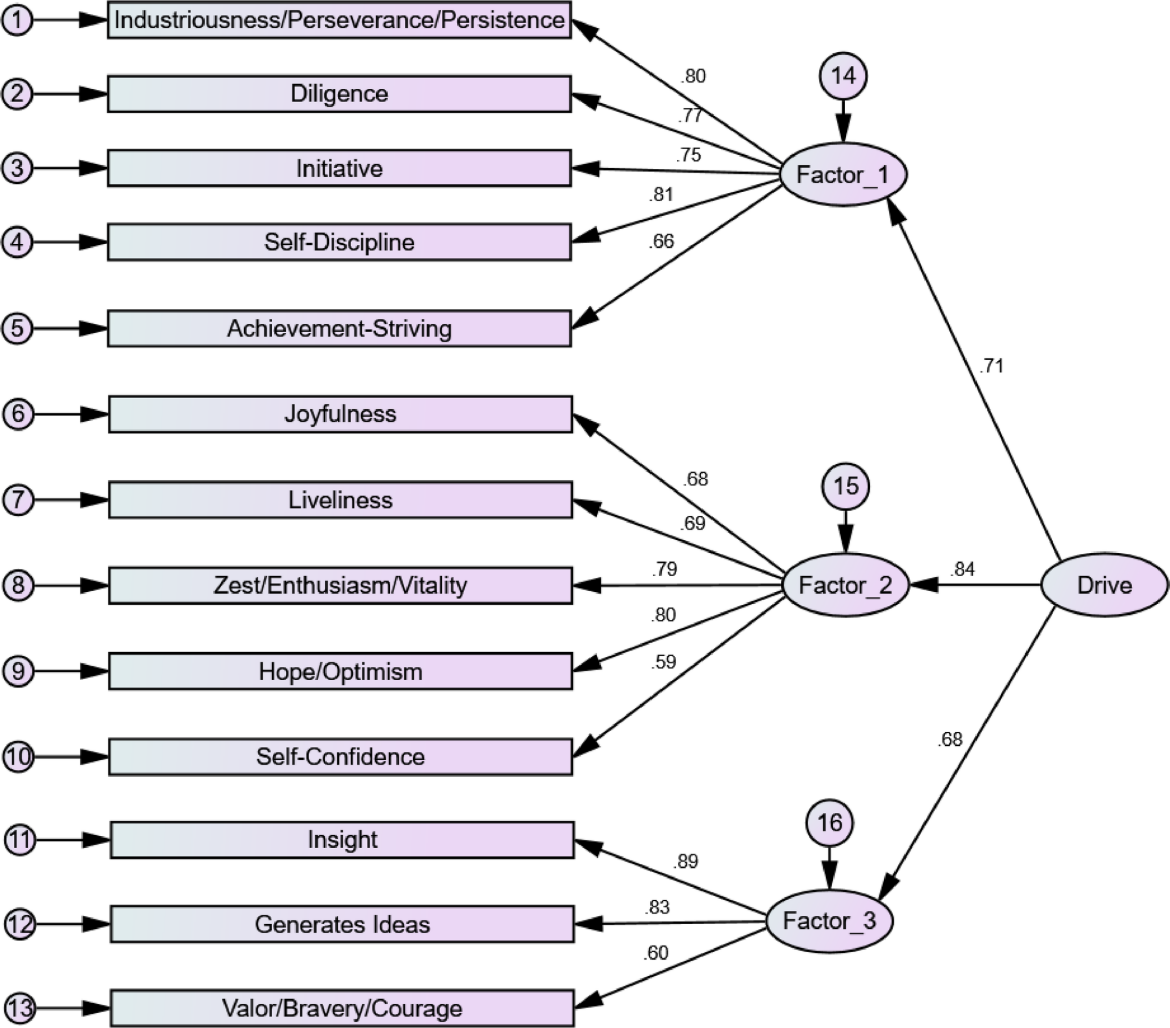
Confirmatory Factor Analysis results for the Drive: International Personality Item Pool version in the Eugene-Springfield community sample (*N* = 496).

The model extracted from the Online and British samples was tested in the Eugene-Springfield community sample. As shown in Fig 1, the pattern of loadings supports the three-factor structure and the theoretical hierarchical structure, with high loadings of the first-order factor on drive and consistent loadings of facets on the first-order factors. However, the model only approached a satisfactory level of fit to the data, χ^2^(64) = 521.53, *p* < .001, CFI = .86, RMSEA = .12, SRMR = .08. No paths were low enough to consider any facets for deletion (e.g., the lowest pointed from the second factor to self-confidence at .59).

Target rotation was performed as an additional check, using the data from the larger of the two exploratory samples as ‘norms’. These results are show in Table 5. Factor congruence coefficients (proportionality coefficients) were consistently high at .99, .94, and .96. By and large, the facet-factor correspondence was also replicated, as indicated by the Square Root of Mean Square Difference values included within table. As in the Online and British samples, factor intercorrelations were moderate at .56 (Factors 1 and 2), .35 (Factors 1 and 3), and .53 (Factors 2 and 3; *p* < .001 for all) in the Eugene-Springfield community sample.

**Table 5:** Factor Loadings after Target Rotation and Congruence Coefficients in the Eugene-Springfield Community Sample.

Facet	Factor loading			Square Root of Mean Square Difference
	1	2	3	
Diligence	**0.69**	0.15	-0.11	0.20
Initiative	**0.88**	-0.19	-0.01	0.11
Self-discipline	**0.87**	-0.10	0.02	0.10
Ind./persev./persis.	**0.72**	0.14	-0.10	0.11
Achievement-striving	**0.53**	0.05	0.20	0.14
Joyfulness	-0.08	**0.73**	0.05	0.25
Hope/optimism	-0.06	**0.93**	-0.10	0.09
Liveliness	0.13	**0.59**	0.00	0.14
Zest/enthusiasm/vitality	0.09	**0.78**	-0.08	0.09
Self-confidence	0.16	**0.28**	0.37	0.23
Insight	-0.08	0.07	**0.86**	0.06
Generates ideas	0.02	-0.06	**0.92**	0.06
Valour/bravery/courage	0.07	0.25	**0.40**	0.07
**FCC**	0.99	0.94	0.96	

*N* = 496. Target loadings are shown in boldface. Ind./persev./persis. = Industriousness/perseverance/persistence; FCC = factor congruence coefficient.

Factor analysis results support a superordinate construct, featuring a hierarchical three-factor structure. The derived factors reflect the “ABCs” of individual differences [58], indicating that drive has affective, behavioural, and cognitive aspects that all need to be considered in an attempt to assess the construct comprehensively. The first factor comprises the facets of industry/perseverance/persistence, self-discipline, diligence, initiative, and achievement-striving; it represents the behavioural aspect of drive. The second factor comprises the facets joyfulness, hope/optimism, liveliness, zest/enthusiasm/vitality, and self-confidence; it represents the affective aspect of drive. The third factor consists of generates ideas, insight, and valour/bravery/courage; it represents the cognitive aspect of drive.

#### Composite internal reliability

Calculated on the basis of Drive:IPIP facet scales, Cronbach’s alpha was fully adequate and stable across the four samples at .95 (Online sample), .91 (Eugene-Springfield community sample), .94 (British sample), and .93 (Norwegian sample). Likewise, Drive:S facet estimates were internally consistent, reaching a level similar to that of the Drive:IPIP scores across samples; Cronbach’s alpha was at .91 (Online sample), .89 (British sample), and .89 (Norwegian sample). Even when given by informants, Drive:S estimates reached decent consistencies in the Online sample (α = .89) and in the British sample (α = .84). These results attest to the internal consistency reliability of the facets. They also suggest that the use of Likert versus visual analogue scale format has little influence on internal reliability of single-item ratings of the facets.

#### Measurement congruence and cross-informant coefficients

As shown in Table 6, bivariate correlations between the Drive:S and Drive:IPIP composites were similar in the Online (*r* = .81) and Norwegian samples (*r* = .83), but somewhat weaker in the British sample (*r* = .73). Although consistently strong in magnitude, these less than “perfect” coefficients are not surprising. The Drive:S facet scores are based on a proxy method of measurement (direct estimates), which may be more susceptible to measurement error than a representative set of items. The results are certainly more than encouraging for the Drive:S to qualify as a brief measure of drive. Drive:S informant-ratings correlated moderately with Drive:IPIP self-ratings in the Online sample (*r* = .47) and with Drive:S self-ratings in both samples (*r* = .36 and .53); they correlated weakly with participants’ Drive:IPIP self-ratings in the British sample (*r* = .22).

**Table 6:** Descriptive Statistics and Bivariate Correlations among Drive:IPIP, Drive:S, and Motivation Perceptions in the Online, British, and Norwegian Samples.

Sample	Variable	*N*	*M*	*SD*	Skewness	Kurtosis	Drive:IPIP	Drive:S	Perception	Drive:S (informant)	Perception (informant)
Online sample	Drive:IPIP	362	3.43	0.58	-0.33	-0.11	—				
	Drive:S	351	61.10	16.63	-0.49	0.22	0.81***	—			
	Perception	351	61.50	25.22	-0.56	-0.41	0.69***	0.77***	—		
	Drive:S (informant)	46	63.84	15.37	-0.17	-0.07	0.47***	0.53***	0.35*	—	
	Perception (informant)	46	63.91	22.66	-0.14	-0.81	0.38**	0.41**	0.30*	0.78***	—
British sample	Drive:IPIP	241	3.37	0.49	-0.01	-0.10	—				
	Drive:S	233	61.28	14.36	-0.43	0.31	0.73***	—			
	Perception	233	63.18	21.35	-0.41	-0.38	0.62***	0.69***	—		
	Drive:S (informant)	101	72.01	11.05	-0.23	-0.21	0.22*	0.36***	0.28**	—	
	Perception (informant)	101	76.28	19.57	-1.16	1.33	0.26**	0.37***	0.39***	0.57***	—
Norwegian sample	Drive:IPIP	142	3.59	0.49	-0.67	1.19	—				
	Drive:S	142	4.94	0.88	-0.74	1.38	0.83***	—			
	Perception	142	5.26	1.34	-0.97	0.97	0.67***	0.71***	—		

Drive:S response scale differed for the Norwegian sample (see Method section). Drive:IPIP = Drive: International Personality Item Pool version; Drive:S = Drive: Short.

*p < .05.

**p < .01.

***p < .001.

## Study 3: External Validity

The external validity, or relationships with other constructs [35], was the focus of Study 3. Additional data obtained or available from the Study 2 samples were used to examine the convergent validity (hypothesised correlations with current motivational states, Neuroticism, Conscientiousness, Ambition, Service Potential, Clerical Potential, and Managerial Potential), discriminant validity (hypothesised non-significant or weak correlations with Agreeableness, Service Orientation, and School Success, as well as a distinct factor when factor-analysed jointly with personality), concurrent validity (hypothesised correlations with global self- and informant-perceptions of motivation, depression, dietary health behaviour, exercise, substance use/smoking, and work avoidance), and incremental validity (hypothesised explanatory effects on depression, dietary health behaviour, exercise, substance use/smoking, and work avoidance, controlling for personality).

### Method

#### Samples and variables

The British and Norwegian samples completed a state measure of motivation level (while state motivation is situation- and time-dependent, stable individual differences in drive should also play a role). Added to the data collection with a slight delay, only 175 participants of the British sample (72.6%) completed the measure. Additional constructs assessed in the Eugene-Springfield community sample could be used to further examine convergent validity: Conscientiousness, Neuroticism, Ambition, Service, Clerical, and Managerial Potential. The role of specific personality constructs was emphasised in the introduction, while ambition is synonymous with drive. These constructs should show sizable correlations with drive. Service, clerical, and managerial potential are aggregate constructs that implicate some degree of drive, next to a host of other attributes; they should show at least significant correlations with drive.

Conceptually distinct personality constructs were also assessed in the Eugene-Springfield community sample: Agreeableness, Service Orientation, Reliability, and School Success. School Success is a scale measuring the degree to which a person enjoys academic activities and values educational achievement for its own sake [59]; it resembles particular motivators rather than drive. The Drive:IPIP should show at most weak associations with these three constructs.

Global self- and informant-perceptions of motivation were measured in the Online, British, and Norwegian samples. Since informant data were obtained several months after collecting the participant data, these informant-perceptions also provide a gauge for predictive validity. Relevant criteria available or derived from the Eugene-Springfield community sample were depression, dietary health behaviour, exercise, and work avoidance, most of which are universally important and implicate some degree of drive. Incremental validity analyses were conducted on these criteria, controlling for higher-order personality dimensions. Partialling out the variance of personality is important for demonstrating the value of drive, given some overlap; both should explain unique variance these criteria. For work avoidance, additional analyses controlled for the effects of a second, occupational personality measure.

#### Measures

The British and Norwegian samples completed the Motivation and Energy Inventory (MEI) [23], a 27-item measure of three highly interrelated factors (Mental Energy, Social Motivation, and Physical Energy) that were combined into a total state motivation score. Most items are measured on a 6- or 7-point Likert scale (frequency type), ranging from *Never* to *Every day or nearly every day* (e.g., “During the past 4 weeks, how often did you feel enthusiastic when you began your day?”), *Never or None of the time* to *All of the time* (e.g., “During the past 4 weeks, how often did you avoid social conversations with others?”), or *Never* to *At least 7 times a week* (e.g., During the past 4 weeks, how often did you engage in recreational activities or hobbies?). Another six items are “to what extent” questions and have a 5-point Likert scale, ranging from *Not at all interested* to *Extremely interested* (e.g., “During the past 4 weeks, to what extent were you interested in learning or trying new things?”). Internal reliabilities are shown in Table 7.

**Table 7:** Descriptive Statistics and Bivariate Correlations of the Drive:IPIP and Drive:S with MEI Scales in the British and Norwegian Samples.

Sample	Variable	*M*	*SD*	Skewness	Kurtosis	Drive:IPIP	Drive:S	Mental Energy	Physical Energy	Social Motivation	Total score
British sample (*N* = 175)	Mental Energy	35.77	9.99	-0.55	1.00	0.57	0.46	-0.87			
	Physical Energy	20.47	7.06	-0.27	-0.23	0.42	0.38	0.56	-0.82		
	Social Motivation	27.20	7.82	-0.27	-0.16	0.36	0.31	0.25	0.44	-0.83	
	Total score	83.44	19.39	-0.60	1.01	0.59	0.50	0.82	0.83	0.69	-0.90
Norwegian sample (*N* = 142)	Mental Energy	43.00	11.45	-1.34	2.00	0.61	0.45	-0.91			
	Physical Energy	22.63	8.30	-0.52	-0.23	0.54	0.42	0.71	-0.86		
	Social Motivation	24.96	8.07	-0.62	0.29	0.61	0.52	0.63	0.66	-0.81	
	Total score	90.78	24.10	-1.01	1.19	0.68	0.54	0.91	0.88	0.85	-0.94

Cronbach’s alphas for the MEI scores are presented in parentheses along the diagonal. Correlations are mostly significant at *p* < .001. Drive:S response scale differed between the two samples (see Method section). Drive:IPIP = Drive: International Personality Item Pool version; Drive:S = Drive: Short; MEI = Motivation and Energy Inventory [23].

The Online, British, and Norwegian samples answered an additional item (“motivation”) with the facet estimates, to be used separately as a criterion (signifying motivation perception). Hence, the Online and British samples gave their global motivation perception on a percentage-based visual analogue scale, while the Norwegian sample used a 7-point Likert scale, as described and explained in Study 2.

The Eugene-Springfield community sample completed the measures described in the remaining paragraphs of this section. The NEO Personality Inventory–Revised (NEO PI-R) [60] and Hogan Personality Inventory (HPI) [59] were used as measures of personality traits. Variables used from these two measures were the Big Five domains and 30 facets of the NEO PI-R as well as the seven primary scales (Adjustment, Ambition, Sociability, Likability, Prudence, Intellectance, and School Success) and six occupational scales (Service Orientation, Stress Tolerance, Reliability, Clerical Potential, Sales Potential, Managerial Potential) of the HPI. The five NEO PI-R domains and their facets comprise a total of 240 items (48 per domain, 8 per facet), which are responded to on a 5-point Likert scale, ranging from *Strongly Disagree* to *Strongly Agree*. In contrast, the HPI uses true-or-false items, ranging in number from 14 to 37 for the primary scales and from 14 to 67 for the occupational scales. While internal reliabilities could not be calculated, both measures consistently produce scores of high internal reliability [59,60], and adequate levels of internal reliability for these data were reported in previous publications, listed on the IPIP website.

Depression was measured using a modified 24-item version of the Center for Epidemiologic Studies Depression Scale [61], which was extended by a few items. This measure was developed specifically to assess depressive symptomatology in non-clinical populations. Respondents indicate how frequently they experienced a range of depressive symptoms during the past week (e.g., “I had a poor appetite”). Items were responded to on a 5-point Likert scale, ranging from 1 (*not at all past week*) to 5 (*most or all of the time*). Internal reliability was adequate (Cronbach’s α = .81).

Healthy diet was measured on the basis of 49 self-report items [62]. Twenty of the items ask about specific health food practices (e.g., “When eating red meat, trim all visible fat?”, “Have a vegetarian dinner?”) and were rated on a 5-point Likert scale, ranging from 1 (*usually or always*) to 5 (*N/A*). The other 29 items ask about the frequency of intake of various food items or liquids (e.g., French fries, Oat bran or wheat germ, 1% or skim milk), using a 5-point scale of 1 (*1< once/month*) to 5 *(≥ 5 times/week*). Following [62], a total “healthy diet” composite was derived from all 49 items. Cronbach’s alpha was .68 (ω = .89).

Exercise, substance use/smoking, and work avoidance were derived from relevant items included in the “Behavioral Report Form” [63], described in a technical report by the Oregon Research Institute [64]. The items describe past behaviour and are rated on a 5-point Likert frequency scale from 1 (*never in my life*) to 5 *(≥ 15 times in past*). A total of 398 items were screened for construct-relevant criteria, resulting in three different clusters of items: exercise (five items), substance use/smoking (22 items), and work avoidance (five items), all of which involve effort to either engage in adaptive behaviour or abstain from maladaptive behaviour.

Each of these item clusters was submitted to a Principal Component Analysis to extract a common dimension, representative of these categories. All five exercise items (e.g., “Participated in an exercise program”) loaded on a single dimension and indicated good reliability (α = .83). The 22 substance use/smoking items (e.g., “smoked tobacco”, “had a hangover”) shared a common factor, but five items (aspirin or ibuprofen, antacids, tranquilising pills, laxative, no-doz or other stay-awake pills) did not load well (≤ .30) and were dropped, due to their distinct nature; items with loadings greater than .30 represented alcohol use, (hard) drug use, or smoking and were internally consistent (α = .88). Two components emerged from the five work avoidance items. However, the second component was selected, as it represented the theoretical criterion: positive loadings of work avoidance behaviours (“was late for work” and “called in sick to work because I was too tired to get up”) and negative loadings of work engagement behaviours (“stayed late at work”, “went to work”, and “stayed away from a social event in order to finish some work”); the first component had positive loadings of all five items, which is more indicative of work intensity. McDonald’s omega for this second component was .79.

#### Statistical analyses

Bivariate correlations were used to examine the convergent, discriminant, and concurrent validities of the Drive:IPIP. Using principal axis factoring, a joint Promax-rotated factor analysis (delta = 4) of the 13 Drive:IPIP facet scales and 30 NEO PI-R facet scales was conducted to examine the level of support for the hypothesised distinct factor within personality-factor space. Incremental validity was assessed via hierarchical regressions of each criterion on (a) higher-order personality dimensions at Step 1 and (b) the Drive:IPIP total composite at Step 2. The NEO PI-R domains functioned as control variables for all criteria; the HPI primary and occupational scales were used separately as additional control variables for work avoidance, given their occupational focus. Only the employed part of the sample (full- or part-time) was used for the analyses involving work avoidance.

### Results and Discussion

#### Convergent and discriminant validity

Bivariate correlations for both Drive forms and the MEI scales are shown in Table 7. These were consistently within a moderate range in both the British sample and the Norwegian sample, although slightly higher for the Drive:IPIP than for the Drive:S and, as can be expected, highest with the MEI total score. The magnitude of associations speaks to the convergent validity of the two Drive versions, but without indicating redundancy. The MEI is a clinically slanted measure of current motivational levels (i.e., states), whereas the Drive:IPIP and Drive:S are general and based on a comprehensive sampling of drive facets.

Intercorrelations among the Drive:IPIP, NEO PI-R domains, and HPI scales are shown in Table 8. Consistent with the stated predictions, the Drive:IPIP did not correlate with Agreeableness, an interpersonal style that is conceptually distinct from drive, whereas it correlated strongly with Conscientiousness. Conscientiousness was previously emphasised as comprising several motivational traits [30,65], and a few of the Drive:IPIP facets (achievement-striving, self-discipline, diligence) derive from this domain. The observed moderate association with Neuroticism also is in accordance with previous findings [66].

**Table 8:** Descriptive Statistics and Intercorrelations of Drive:IPIP, NEO PI-R Domains, and HPI Primary and Occupational Scales in the Eugene-Springfield Community Sample.

Variable	1	2	3	4	5	6	7	8	9	10	11	12	13	14	15	16	17	18	19
1. Drive:IPIP	—																		
2. Neuroticism	-0.57***	—																	
3. Extraversion	0.56***	-0.28***	—																
4. Openness	0.26***	-0.03	0.38***	—															
5. Agreeableness	0.03	-0.22***	0.02	0.02	—														
6. Conscientiousness	0.61***	-0.46***	0.18***	-0.14**	0.13**	—													
7. Adjustment	0.37***	-0.73***	0.16***	0.02	0.31***	0.23***	—												
8. Ambition	0.69***	-0.54***	0.56***	0.21***	-0.12*	0.40***	0.45***	—											
9. Sociability	0.29***	-0.03	0.62***	0.42***	-0.27***	-0.07	0.02	0.45***	—										
10. Likability	0.31***	-0.27***	0.44***	0.21***	0.48***	0.09	0.42***	0.29***	0.16***	—									
11. Prudence	0.11*	-0.26***	-0.07	-0.33***	0.46***	0.42***	0.34***	0.03	-0.40***	0.26***	—								
12. Intellectance	0.34***	-0.16***	0.24***	0.52***	-0.19***	0.07	0.12**	0.38***	0.47***	0.04	-0.25***	—							
13. School Success	0.22***	-0.12**	0.07	0.23***	-0.07	0.11*	0.11*	0.22***	0.12*	0.01	0.05	0.32***	—						
14. Service Orientation	0.19***	-0.41***	0.07	0.04	0.53***	0.09	0.63***	0.14**	-0.13**	0.62***	0.43***	-0.03	0.00	—					
15. Stress Tolerance	0.50***	-0.76***	0.23***	0.07	0.13**	0.30***	0.89***	0.58***	0.10*	0.31***	0.22***	0.21***	0.16***	0.46***	—				
16. Reliability	0.06	-0.38***	-0.07	-0.25***	0.46***	0.24***	0.59***	0.07	-0.33***	0.33***	0.70***	-0.20***	0.02	0.41***	0.36***	—			
17. Clerical Potential	0.55***	-0.60***	0.45***	0.19***	0.05	0.30***	0.64***	0.76***	0.31***	0.38***	0.17***	0.32***	0.22***	0.31***	0.72***	0.25***	—		
18. Sales Potential	0.46***	-0.22***	0.69***	0.49***	-0.17***	0.03	0.19***	0.65***	0.88***	0.38***	-0.35***	0.58***	0.18***	0.03	0.28***	-0.27***	0.48***	—	
19. Managerial Potential	0.67***	-0.55***	0.46***	0.14**	0.01	0.47***	0.53***	0.86***	0.29***	0.31***	0.29***	0.33***	0.38***	0.21***	0.64***	0.27***	0.82***	0.45***	—
***N***	496	475	475	475	475	475	476	476	476	476	476	476	476	476	476	476	476	476	476
***M***	3.72	80.09	105.21	113.27	125.57	124.90	24.15	20.76	10.55	18.33	19.70	13.32	8.33	9.50	18.17	11.57	17.06	36.60	27.74
***SD***	0.43	23.71	20.24	21.63	16.91	19.36	6.87	5.72	4.86	3.21	4.10	4.41	3.20	2.47	4.83	3.45	3.82	9.30	5.19
**Skewness**	-0.49	0.51	-0.29	-0.12	-0.52	-0.42	-0.48	-0.76	0.17	-1.44	-0.45	-0.15	-0.44	-0.48	-0.74	-0.41	-0.29	-0.18	-0.67
**Kurtosis**	0.89	0.26	0.07	-0.07	0.61	0.86	-0.37	0.24	-0.59	2.65	-0.07	-0.50	-0.48	-0.22	-0.20	-0.55	-0.38	-0.47	0.61

*N* = 458. Drive:IPIP = Drive: International Personality Item Pool version; NEO PI-R = NEO Personality Inventory–Revised [60]; HPI = Hogan Personality Inventory [59].

*p < .05.

**p < .01.

***p < .001

Concerning the HPI, the highest correlation was observed for Ambition, which is conceptually very similar to the behavioural factor of drive. Moderate associations with Adjustment and Intellectance provide further support for the measure’s convergent validity, with both entailing some degree of drive; Adjustment bears on the behavioural, cognitive, and affective factors in the derived structural model, whereas Intellectance resembles the cognitive and behavioural factors. The weak association with Prudence is not very surprising. Although this variable was initially identified as a potential facet, it was eventually removed from the representation due to weak empirical fit. Also in line with hypothesised associations are moderate correlations observed for Clerical Potential, Sales Potential, and Managerial Potential (indicative of convergent validity), as well as weak and non-significant correlations for Service Orientation, School Success, and Reliability (indicative of discriminant validity).

Promax-rotated factor analysis results are shown in Table 9. Whereas Eigenvalues and scree plot pointed to seven components, parallel analysis extracted only six. The six-factor solution was used, since it accommodates the five personality domains and drive. The pattern of loadings clearly supports the Big Five plus one additional component, representing drive as a distinct dimension. The cross-loadings of certain drive facets on the Big Five domains substantiate the conceptualisation of drive as a construct that permeates personality-factor space. Conversely, there were no noteworthy loadings (≥ .30) of the 30 NEO PI-R facets on the drive component. Its emergence as a distinct dimension corroborates the notion that the construct extends beyond the boundaries of personality, or how people are like, into the space of individual differences theorised to constitute drive.

**Table 9:** Promax-Rotated Pattern Matrix for Drive:IPIP and NEO PI-R Facets in the Eugene-Springfield Community Sample.

Facet	Factor loading					
	C	N	O	A	E	Drive
Self-confidence		0.68				
Zest/enthusiasm/vitality						0.68
Valour/bravery/courage			0.39			
Liveliness						0.47
Joyfulness		0.51			0.37	0.31
Insight			0.71			
Initiative	0.72					
Diligence	0.75					0.33
Generates ideas			0.58			
Ind./persev./persis.	0.75					0.33
Self-discipline	0.78					
Achievement-striving	0.68					
Hope/optimism		0.32				0.52
N1		-0.86				
N2		-0.67		-0.48		
N3		-0.87				
N4		-0.73				
N5		-0.44		-0.32		
N6		-0.70				
E1					0.86	
E2					0.60	
E3				-0.50	0.34	
E4	0.40				0.33	
E5				-0.41	0.32	
E6					0.69	
O1			0.70			
O2			0.75	0.32		
O3		-0.45	0.53		0.38	
O4			0.55			
O5			0.80			
O6			0.51			
A1		0.40		0.35	0.42	
A2				0.64		
A3				0.56	0.56	
A4				0.74		
A5		-0.40		0.53		
A6				0.56		
C1	0.53	0.39				
C2	0.76					
C3	0.69					
C4	0.74					
C5	0.80					
C6	0.50			0.32		
**Eigenvalue**	10.92	5.18	4.02	2.89	1.99	1.45
**% of variance**	25.39	12.04	9.36	6.73	4.64	3.37

*N* = 475. Factor loadings of < .30 are omitted from the table. Drive:IPIP = Drive: International Personality Item Pool version; NEO PI-R = NEO Personality Inventory–Revised [60]. N = Neuroticism, E = Extraversion, O = Openness, A = Agreeableness, C = Conscientiousness.

#### Concurrent and incremental validity

Bivariate correlations of the Drive:IPIP and Drive:S with motivation perceptions are included in Table 6. Correlations of the Drive:IPIP scores were within a moderate range of .62 to .69 for self-perceptions and weak-to-moderate for informant-perceptions (*r* = .26 and .38) of motivation. The same correlations involving the Drive:S were somewhat stronger for self-perceptions (*r* = .69 to .77) and informant-perceptions (*r* = .36 and .53). These systematic differences in magnitude of associations reflect the measurement equivalence of the Drive:S and the criterion in the Online and British samples. Correlations between Drive:S informant-ratings and self-perceptions of motivation were weak in the British sample (*r* = .22) and somewhat larger in the Online sample (*r* = 35). Drive:S informant scores correlated moderately to strongly with informant-perceptions of motivation (*r* = .78 and .57), comparable to the associations of the corresponding self-ratings.

Table 10 shows the bivariate correlations of the Drive:IPIP, NEO PI-R domains, and HPI scales with the five Eugene-Springfield community sample criteria. These were significant and in the expected direction in all cases except for substance abuse, which did not correlate significantly with Drive:IPIP scores.

**Table 10:** Descriptive Statistics and Bivariate Correlations of Criteria with Drive:IPIP, NEO PI-R Domains, and HPI Primary and Occupational Scales in the Eugene-Springfield Community Sample.

Variable	Depression	Exercise	Healthy diet	Substance use/smoking	Work avoidance
Drive:IPIP	-0.37**	0.22**	0.15**	0.00	-0.14*
Neuroticism	0.46**	-0.07	-0.02	0.03	0.10
Extraversion	-0.21**	0.22**	0.04	0.12*	0.09
Openness	0.06	0.11*	0.21**	0.22**	0.17**
Agreeableness	-0.03	-0.05	0.18**	-0.32**	-0.15*
Conscientiousness	-0.19**	0.05	0.10	-0.13**	-0.22**
Adjustment	—	—	—	—	-0.15*
Ambition					0.00
Sociability					0.15*
Likability					-0.03
Prudence					-0.21**
Intellectance					0.05
School Success					-0.04
Service Orientation					-0.18**
Stress Tolerance					-0.10
Reliability					-0.18**
Clerical Potential					-0.01
Sales Potential					0.14*
Managerial Potential					-0.10
***N***	469	477	398	454	256
**Skewness**	1.58	0.20	-0.01	0.36	0.18
**Kurtosis**	2.93	-1.15	-0.43	0.03	-0.50

Drive:IPIP = Drive: International Personality Item Pool version; NEO PI-R = NEO Personality Inventory–Revised [60]; HPI = Hogan Personality Inventory [59].

*p < .05.

**p < .001.

Incremental validity analyses for the Eugene-Springfield community sample criteria are shown in Table 11 (whole sample) and Table 12 (working portion of the sample). The Drive:IPIP explained unique variance beyond the Big Five personality traits in all criteria, including substance abuse. The criterion variance explained was not particularly large for any of the predictors, indicating that variables other than psychological constructs and individual differences (external factors) possibly carry more weight. The significant explanatory effect of the Drive:IPIP on substance abuse suggests that drive has desirable and undesirable effects on substance abuse that cancel each other out to an overall non-significant bivariate correlation. As a result of “trying hard” in life, people who are very motivated may need to cope with the ensuing stress and, consequently, may resort to maladaptive coping strategies, such as drinking and smoking. On the other hand, drive may also lead people to abstain from substance use/smoking, as indicated by the significant explanatory effect of drive when controlling for the Big Five domains.

**Table 11:** Hierarchical Regression Analyses Predicting Criteria with the NEO PI-R Domains (Step 1) and the Drive:IPIP (Step 2) in the Eugene-Springfield Community Sample.

Step	Depression			Exercise			Healthy diet			Substance use/smoking		
1	*F*(5,444) = 28.06**			*F*(5,451) = 5.04**			*F*(5,377) = 7.39**			*F*(5,429) = 17.05**		
	Δ*R*^2^ = 0.240**, *R*^2^_Adj_ = 0.232			Δ*R*^2^ = 0.053**, *R*^2^_Adj_ = 0.042			Δ*R*^2^ = 0.089**, *R*^2^_Adj_ = 0.077			Δ*R*^2^ = 0.166**, *R*^2^_Adj_ = 0.156		
2	*F*(6,443) = 28.18**			*F*(6,450) = 6.20**			*F*(6,376) = 7.09**			*F*(6,428) = 15.27**		
	Δ*R*^2^ = 0.036**, *R*^2^_Adj_ = 0.266			Δ*R*^2^ = 0.023**, *R*^2^_Adj_ = 0.064			Δ*R*^2^ = 0.012*, *R*^2^_Adj_ = 0.087			Δ*R*^2^ = 0.011*, *R*^2^_Adj_ = 0.165		
**Step 2 predictors**	**β**	**Tolerance**	**VIF**	**β**	**Tolerance**	**VIF**	**β**	**Tolerance**	**VIF**	**β**	**Tolerance**	**VIF**
N	0.37**	0.62	1.62	0.05	0.61	1.63	0.13*	0.60	1.65	-0.12*	0.61	1.65
E	-0.03	0.63	1.59	0.11*	0.62	1.61	-0.12	0.63	1.60	0.12*	0.62	1.62
O	0.18**	0.74	1.35	-0.02	0.74	1.35	0.19**	0.72	1.39	0.23**	0.73	1.37
A	0.04	0.91	1.10	-0.03	0.92	1.09	0.19**	0.89	1.12	-0.35**	0.92	1.08
C	0.21**	0.50	1.98	-0.11	0.50	2.01	0.05	0.49	2.04	-0.02	0.50	1.99
Drive:IPIP	-0.33**	0.33	3.07	0.27**	0.32	3.13	0.20*	0.31	3.26	-0.18*	0.32	3.16

NEO PI-R = NEO Personality Inventory–Revised [60]; Drive:IPIP = Drive: International Personality Item Pool version; VIF = variance inflation factor, N = Neuroticism, E = Extraversion, O = Openness, A = Agreeableness, C = Conscientiousness.

*p < .05.

**p < .001.

As regards work avoidance, the results show that the Drive:IPIP predicts unique criterion variance irrespective of whether the NEO PI-R domains, HPI primary scales, or HPI occupational scales are controlled for as predictors. Again, the variance was not very large for any measure, but the incremental variance explained by the Drive:IPIP went up to 6.1% (vis-à-vis the NEO PI-R) and the Drive:IPIP showed the largest beta weight when included with the NEO PI-R domains or HPI primary scales.

**Table 12:** Hierarchical Regression Analysis Predicting Work Avoidance with the NEO PI-R Domains (Step 1a), HPI Primary Scales (Step 1b), or HPI Occupational Scales (Step 1c) and the Drive:IPIP (Steps 2a, 2b, and 2c) in the Eugene-Springfield Community Sample.

Step	Model summary		
1a	*F*(5,250) = 4.60***, Δ*R*^2^ = 0.084***, *R*^2^_Adj_ = 0.066		
1b	*F*(7,248) = 2.30*, Δ*R*^2^ = 0.061*, *R*^2^_Adj_ = 0.035		
1c	*F*(6,249) = 4.14***, Δ*R*^2^ = 0.091***, *R*^2^_Adj_ = 0.069		
2a	*F*(6,249) = 4.89***, Δ*R*^2^ = 0.021*, *R*^2^_Adj_ = 0.084		
2b	*F*(8,247) = 3.05**, Δ*R*^2^ = 0.029**, *R*^2^_Adj_ = 0.060		
2c	*F*(7,248) = 4.22***, Δ*R*^2^ = 0.016*, *R*^2^_Adj_ = 0.081		
**Step 2 predictors**	**β**	**Tolerance**	**VIF**
**2a**			
Neuroticism	-0.06	0.65	1.53
Extraversion	0.14	0.70	1.42
Openness	0.16*	0.75	1.34
Agreeableness	-0.13*	0.91	1.10
Conscientiousness	-0.08	0.56	1.79
Drive:IPIP	-0.23*	0.38	2.60
**2b**			
Adjustment	-0.10	0.64	1.57
Ambition	0.13	0.43	2.34
Sociability	0.07	0.54	1.86
Likability	0.07	0.74	1.35
Prudence	-0.15	0.64	1.56
Intellectance	0.02	0.67	1.49
School Success	-0.02	0.85	1.18
Drive:IPIP	-0.23**	0.54	1.86
**2c**			
Service Orientation	-0.14	0.66	1.52
Stress Tolerance	-0.01	0.40	2.48
Reliability	-0.05	0.58	1.71
Clerical Potential	0.22	0.22	4.47
Sales Potential	0.19*	0.52	1.92
Managerial Potential	-0.21	0.24	4.22
Drive:IPIP	-0.17*	0.56	1.80

NEO PI-R = NEO Personality Inventory–Revised [60]; HPI = Hogan Personality Inventory [59]; Drive:IPIP = Drive: International Personality Item Pool version; VIF = variance inflation factor.

*p < .05.

**p < .01.

***p < .001.

Keeping in mind factors that can be expected to attenuate (small sample sizes involving informant-perceptions) or inflate (common-source and method variance) the observed association, these result provide good preliminary support for the concurrent, incremental, and, to some extent, predictive validity of the instrument(s).

## General Discussion

This article explicated the theory of drive and described the development and validation of two measures, one based on multi-item rating scales, the other on single-item ratings. A set of facets systematically derived from the IPIP was used to develop a clean and replicable structural model, comprised of the superordinate construct, three first-order factors (representing affective, behavioural, and cognitive aspects), and 13 facets. The global composite demonstrated convergent and discriminant validity with conceptually similar and distinct personality constructs, respectively, as well as concurrent and incremental validity of relevant criteria, including informant-perceptions.

The results support the validity of drive, as operationalised in the present investigation. In particular, they support the existence of a superordinate construct, which explained a considerable part of the variance in the drive facets (in excess of 75%). This result is in line with the properties of similar constructs, which assume a general factor [23,25] and presumably represent aspects of drive. As previously argued [67] and demonstrated for other constructs, such as trait emotional intelligence [68], the results also illustrate that facet-level personality traits possess and share variance not encompassed by the established personality taxonomies, such as the Big Five domains. One systematic source of variance of certain facet traits appears to be drive, which runs across multiple personality dimensions and beyond.

Although drive naturally permeates general personality dimensions, it is far from redundant with them. For example, the maximum correlation with the Big Five personality traits was between the Drive:IPIP and Conscientiousness at *r* = 61. Drive also explained incremental variance in all criteria examined in the present study when controlling for the Big Five domains and, in the case of work avoidance, also for the HPI scales. Even more convincing is that a joint factor analysis of Drive:IPIP and NEO PI-R facets supported a distinct sixth dimension, distinguished clearly by loadings of drive facets only. In comparison, a correlation of .77 was observed between the similar construct of Grit and Conscientiousness, but the Grit Scale still evidenced incremental validity vis-à-vis the Big Five domains [24,25]. These results strongly support the distinctiveness and utility of drive, relative to established personality dimensions. Accordingly, they reflect the notion that the Five-Factor Model is a suitable taxonomy of motivational traits [10], which seem partially distributed across multiple personality dimensions (if unevenly) and to extend beyond them.

### Implications

To some degree, the field of motivation (from an individual differences and assessment perspective) has been stepping in the dark at a conceptual level in its heavy focus on motivators [1]. While motivators constitute a valid and useful set of attributes, they must not be confounded with drive, as argued in the introduction. Motivators, such as motives, were found to be multidimensional, involving multiple constructs [4–6,27,28], whereas drive represents a single, superordinate construct. A main contribution of the present work lies in the explication of the theory of drive, a construct largely neglected in the scientific literature and supported by the results of this investigation.

The construct of drive has conceptual appeal over cognate, unique-sounding conceptualisations of the target entity. For example, most people have at least a basic idea of what drive means. Drive is a concept that, presumably for good reason, has been part of our common language for quite some time. More importantly, it is grounded in a firm theory and situated appropriately within the context of established individual-differences taxonomies. It permeates the factor space of personality but also transcends personality boundaries to cover the (unique) space of motivational level.

Surprisingly few measures can be found that seem to measure the construct at least partially. These are the MEI [23], the Grit Scale [24,25], and possibly also the Motivation and Engagement Scale [22]. However, these measures have uncertain validity as far as the construct of drive in terms of individual differences is concerned. After all, none of them were developed to measure the construct specifically. The MEI may come closest, but it has a clinical emphasis on recent motivational levels, or motivational states. The model advanced here was not only developed to represent drive explicitly, but it also originates from a comprehensive set of indicator traits, increasing the likelihood of full construct coverage.

As a matter of necessity, much of the article has focused on the theory and measurement of drive. If one accepts the construct and builds adequate measures, the question remains why we should be interested in it beyond theory, measurement, and research. Subject to further research into its role in various domains of life (work, education, health, etc.), several applications can be envisioned in the context of assessment and development, especially in organisational and business contexts. For example, it may be desirable to select “driven” individuals for certain jobs, whereas for other roles, the extremely driven may be quick to leave, seeking to advance and “move on” quickly. One may wish to avoid highly driven candidates for certain roles in order to avoid high staff turnover.

While the primary application of drive would seem to lie in selection, it also is plausible that assessment of the construct has utility in personnel development and organisational psychology, more generally. It may pay to integrate measures of the construct in formative assessments, such as to understand more clearly the reasons behind weak performance and to inform personnel development. In any case, possible development applications will demand further theoretical development and, especially, applied research.

### Limitations and Future Directions

At present, only a single structural model and two measures of the proposed construct exist, although the interpretation of results gathered with related measures through the lens of drive is encouraged. For the field to (re)gain momentum, it will be necessary that alternative measures be systematically devised. On the one hand, a plethora of models and measures often complicates the integration of research findings, particularly where measures vary considerably in scope and focus. Still, convincing and converging results from multiple measures would advance theory and support the value of the construct. The key point to be considered is that multiple drive models and measures ought to be grounded in the same general conceptual definition of the construct, especially one that does not overlap with motivators.

Further psychometric efforts are needed to systematically establish the nomological network and criterion validity of drive, involving constructs not considered in the present investigation. Although the construct was defined in detail, any operational vehicle(s) should undergo systematic (convergent and discriminant) validation efforts, ideally within a multitrait-multimethod framework [69]. For example, the distinctiveness between drive and various motivators remains to be ascertained thoroughly. The focus would then naturally shift to the role of the construct in relevant contexts, by examining its predictive effects on objectively assessed real-life criteria (e.g., performance and achievement). The measures developed in this investigation seem trustworthy as operational vehicles for these endeavours.

## Supporting Information

S1 Appendix Drive: International Personality Item Pool version (Drive:IPIP), as used in Studies 2 and 3. (DOCX)

S2 Appendix Drive: Short (Drive:S). (DOCX)

S1 Data All data files. (ZIP)
