# Supplementary material for: Drive: Theory and Construct Validation
Source: PLoS One. 2016 Jul 13;11(7):e0157295. doi: 10.1371/journal.pone.0157295 (PMC4943631; doi:10.1371/journal.pone.0157295)
Supplement: S1 Appendix — (DOCX) [file pone.0157295.s001.docx]

Appendix 1:

Drive: International Personality Item Pool version (Drive:IPIP), as used in Studies 2 and 3.

**Self-confidence**

| + keyed | Think highly of myself.  Know immediately what to do. |
| --- | --- |
|  |  |
|  |  |
| – keyed | Have a low opinion of myself.  Am easily intimidated.  Feel threatened easily. |
|  |  |
|  |  |

**Temperance**

| + keyed | Rarely overindulge.  Keep my promises. |
| --- | --- |
|  |  |
|  |  |
| – keyed | Change my mood a lot.  Am guided by my moods.  Am preoccupied with myself.  Grumble about things.  Suddenly lose interest.  Say inappropriate things.  Love to come up with objections. |
|  |  |
|  |  |
|  |  |
|  |  |
|  |  |
|  |  |

**Zest/enthusiasm/vitality**

| + keyed | Prefer to participate fully rather than view life from the sidelines.  Don't approach things halfheartedly.  Love what I do.  Look forward to each new day.  Can’t wait to get started on a project.  Can hardly wait to see what life has in store for me in the years ahead.  Awaken with a sense of excitement about the day's possibilities. |
| --- | --- |
|  |  |
|  |  |
|  |  |
|  |  |
|  |  |
|  |  |
|  |  |
| - keyed | Dread getting up in the morning.  Don't have much energy. |
|  |  |

**Valor/bravery/courage**

| + keyed | Have taken frequent stands in the face of strong opposition.  Don't hesitate to express an unpopular opinion.  Call for action while others talk.  Can face my fears.  Speak up in protest when I hear someone say mean things.  Am a brave person. |
| --- | --- |

**Liveliness**

| +keyed | Maintain high energy throughout the day.  Have great stamina.  Am usually active and full of energy.  Smile a lot.  Feel healthy and vibrant most of the time.  Laugh a lot.  Feel that I have a lot of inner strength. |
| --- | --- |
|  |  |
|  |  |
|  |  |
|  |  |
|  |  |
|  |  |
|  |  |
| - keyed | Tire out quickly. |

**Insight**

| + keyed | Come up with something new. |  |
| --- | --- | --- |
|  | Throw a new light on the situation. | |
|  | Come up with alternatives. | |
|  | Put a new perspective on things. | |
|  | Have a vivid imagination. | |
|  |  | |
| – keyed | Consider myself an average person. | |

**Initiative**

| + keyed | Get things done quickly.  Get to work at once.  Finish tasks quickly.  Start tasks right away. |
| --- | --- |
|  |  |
| – keyed | Put off unpleasant tasks. |

**Diligence**

| +keyed | Push myself very hard to succeed.  Get started quickly on doing a job.  Am exacting in my work. |
| --- | --- |
| - keyed | Stop when work becomes too difficult.  Quickly lose interest in the tasks I start. |

**Deliberateness**

| + keyed | Choose my words with care.  Take care of my own affairs.  Remain calm under pressure. |
| --- | --- |
|  |  |
|  |  |
|  |  |
|  |  |
| – keyed | Like to act on a whim.  Rush into things.  Act quickly without thinking. |

**Competitive**

| + keyed | Accept challenging tasks.  Am good at many things. |
| --- | --- |
| – keyed | Am not highly motivated to succeed.  Do just enough work to get by.  Undertake few things on my own. |
|  |  |
|  |  |

**Experience-seeking**

| + keyed | Prefer variety to routine.  Seek adventure.  Try out new things. |
| --- | --- |
|  |  |
|  |  |
|  |  |
| – keyed | Would describe my experiences as somewhat dull.  Dislike new foods. |
|  |  |
|  |  |

**Generates ideas**

| + keyed | Am full of ideas.  Have excellent ideas.  Quickly think up new ideas. |
| --- | --- |
|  |  |
|  |  |
|  |  |
| – keyed | Do not have a good imagination.  Can't come up with new ideas. |
|  |  |

**Prudence**

| +keyed | Avoid mistakes.  Make plans and stick to them.  Do things according to a plan. |
| --- | --- |
|  |  |
|  |  |
|  |  |
| - keyed | Do things without thinking of the consequences.  Act impulsively when something is bothering me.  Make careless mistakes.  Make a fool of myself. |
|  |  |
|  |  |
|  |  |

**Resourcefulness**

| + keyed | Can handle complex problems.  Face problems directly.  Formulate ideas clearly. |
| --- | --- |
|  |  |
|  |  |
|  |  |
|  |  |
|  |  |
| – keyed | Wait for others to lead the way.  Can't make up my mind.  Panic easily. |
|  |  |
|  |  |

**Self-control**

| + keyed | Am not easily affected by my emotions.  Never spend more than I can afford.  Experience very few emotional highs and lows. |
| --- | --- |
|  |  |
|  |  |
|  |  |
| – keyed | Act wild and crazy.  Demand attention.  Do crazy things.  Use flattery to get ahead.  Use swear words.  Make a lot of noise. |
|  |  |
|  |  |
|  |  |
|  |  |
|  |  |
|  |  |

**Ind./persev./persis.**

| + keyed | Don't quit a task before it is finished.  Am a goal-oriented person.  Finish things despite obstacles in the way.  Am a hard worker.  Don't get sidetracked when I work. |
| --- | --- |
|  |  |
|  |  |
|  |  |
|  |  |
|  |  |
| - keyed | Don't finish what I start.  Do not tend to stick with what I decide to do. |
|  |  |
|  |  |

**Activity-level**

| + keyed | Can manage many things at the same time.  Am always busy.  Do a lot in my spare time.  Am always on the go.  Accomplish a lot of work. |
| --- | --- |
|  |  |
|  |  |
|  |  |
|  |  |
|  |  |
| – keyed | Have a slow pace to my life. |

**Adventurousness**

| + keyed | Like to visit new places.  Interested in many things.  Like to begin new things. |
| --- | --- |
|  |  |
|  |  |
|  |  |
|  |  |
| – keyed | Prefer to stick with things that I know.  Dislike changes.  Am a creature of habit.  Am attached to conventional ways. |
|  |  |
|  |  |
|  |  |

**Self-discipline**

| + keyed | Get chores done right away.  Am always prepared.  Carry out my plans. |
| --- | --- |
|  |  |
|  |  |
|  |  |
|  |  |
|  |  |
| – keyed | Waste my time.  Postpone decisions. |
|  |  |

**Achievement-striving**

| + keyed | Go straight for the goal.  Turn plans into actions.  Do more than what's expected of me.  Set high standards for myself and others.  Demand quality. |
| --- | --- |
|  |  |
|  |  |
|  |  |
|  |  |
|  |  |
|  |  |
|  |  |
| – keyed | Put little time and effort into my work. |

**Hope/optimism**

| +keyed | Look on the bright side.  Can find the positive in what seems negative to others.  Remain hopeful despite challenges.  Will succeed with the goals I set for myself.  Think about what is good in my life when I feel down. |
| --- | --- |
|  |  |
|  |  |
|  |  |
|  |  |
|  |  |
| -keyed | Expect the worst.  Have no plan for my life five years from now.  Am not confident that my way of doing things will work out for the best. |
|  |  |

**Competence**

| + keyed | Like to solve complex problems.  Can perform a wide variety of tasks.  Know how to apply my knowledge.  Meet challenges. |
| --- | --- |
|  |  |
|  |  |
|  |  |
|  |  |
|  |  |
|  |  |
| – keyed | Don't put my mind on the task at hand.  Don't see things through. |
|  |  |

**Self-efficacy**

| + keyed | Excel in what I do.  Handle tasks smoothly.  Come up with good solutions.  Know how to get things done. |
| --- | --- |
|  |  |
|  |  |
|  |  |
|  |  |
|  |  |
|  |  |
| – keyed | Misjudge situations.  Don't understand things.  Don't see the consequences of things. |
|  |  |
|  |  |

**Joyfulness**

| + keyed |  | Love life.  Radiate joy.  Feel lucky most of the time.  Just know that I will be a success. |
| --- | --- | --- |
|  |  |  |
|  |  |  |
|  |  |  |
|  |  |  |
| – keyed |  | Am often in a bad mood.  Feel that my life lacks direction.  Have a dark outlook on the future. |
