# Supplementary material for: Drive: Theory and Construct Validation
Source: PLoS One. 2016 Jul 13;11(7):e0157295. doi: 10.1371/journal.pone.0157295 (PMC4943631; doi:10.1371/journal.pone.0157295)
Supplement: S2 Appendix — (DOCX) [file pone.0157295.s002.docx]

Appendix 2:

Drive: Short (Drive:S)

**Instructions:** Below you find a list of characteristics describing people. Please use the rating scale below to describe how much/little of each characteristic you show or possess. Describe yourself as you generally are now, not as you wish to be in the future. Describe yourself as you honestly see yourself, in relation to other people you know of roughly your age. So that you can describe yourself in an honest manner, your responses will be kept in absolute confidence.

| **Item** | **1**  **Very**  **Little** | **2** | **3** | **4** | **5** | **6** | **7**  **Very**  **Much** |
| --- | --- | --- | --- | --- | --- | --- | --- |
| 1. Self-Confidence |  |  |  |  |  |  |  |
| 1. Zest/Enthusiasm/Vitality |  |  |  |  |  |  |  |
| 1. Valor/Bravery/Courage |  |  |  |  |  |  |  |
| 1. Liveliness |  |  |  |  |  |  |  |
| 1. Joyfulness |  |  |  |  |  |  |  |
| 1. Insight |  |  |  |  |  |  |  |
| 1. Initiative |  |  |  |  |  |  |  |
| 1. Diligence |  |  |  |  |  |  |  |
| 1. Generates Ideas |  |  |  |  |  |  |  |
| 1. Industriousness/Perseverance/Persistence |  |  |  |  |  |  |  |
| 1. Self-Discipline |  |  |  |  |  |  |  |
| 1. Achievement-Striving |  |  |  |  |  |  |  |
| 1. Hope/Optimism |  |  |  |  |  |  |  |
